# PCOGR: Phylogenetic COG ranking as an online tool to judge the specificity of COGs with respect to freely definable groups of organisms

**DOI:** 10.1186/1471-2105-5-150

**Published:** 2004-10-15

**Authors:** Florian Meereis, Michael Kaufmann

**Affiliations:** 1 The Protein Chemistry Group, Institute of Neurobiochemistry, Witten/Herdecke University, Stockumer Str. 10, 58448 Witten, Germany

## Abstract

**Background:**

The rapidly increasing number of completely sequenced genomes led to the establishment of the COG-database which, based on sequence homologies, assigns similar proteins from different organisms to clusters of orthologous groups (COGs). There are several bioinformatic studies that made use of this database to determine (hyper)thermophile-specific proteins by searching for COGs containing (almost) exclusively proteins from (hyper)thermophilic genomes. However, public software to perform individually definable group-specific searches is not available.

**Results:**

The tool described here exactly fills this gap. The software is accessible at  and is linked to the COG-database. The user can freely define two groups of organisms by selecting for each of the (current) 66 organisms to belong either to groupA, to the reference groupB or to be ignored by the algorithm. Then, for all COGs a specificity index is calculated with respect to the specificity to groupA, *i. e. *high scoring COGs contain proteins from the most of groupA organisms while proteins from the most organisms assigned to groupB are absent. In addition to ranking all COGs according to the user defined specificity criteria, a graphical visualization shows the distribution of all COGs by displaying their abundance as a function of their specificity indexes.

**Conclusions:**

This software allows detecting COGs specific to a predefined group of organisms. All COGs are ranked in the order of their specificity and a graphical visualization allows recognizing (i) the presence and abundance of such COGs and (ii) the phylogenetic relationship between groupA- and groupB-organisms. The software also allows detecting putative protein-protein interactions, novel enzymes involved in only partially known biochemical pathways, and alternate enzymes originated by convergent evolution.

## Background

The COG-database has become a powerful tool in the field of comparative genomics. The construction of this data base is based on sequence homologies of proteins from different completely sequenced genomes. Highly homologous proteins are assigned to clusters of orthologous groups (COGs) [1,2]. Each of the COGs consists of individual proteins or groups of orthologs from at least 3 lineages and thus corresponds to a conserved domain. The COG collection currently consists of 138,458 proteins, which form 4,873 COGs and comprise 75% of the 185,505 (predicted) proteins encoded in 66 genomes of unicellular organisms [3]. In addition, the database now includes KOGs containing the clusters of seven eukaryotic genomes. The COG database is an ideal source to search for proteins specific to a certain group of organisms. Several such surveys aimed at finding (hyper)thermophile-specific proteins that made use of the COG-database are published. For instance, Forterre detected reverse gyrase as the only hyperthermophile-specific protein [4]. In addition, a survey to find specific genes important for hyperthermophily [5] and a study identifying thermophile-specific proteins [6] are published. However, those studies used rather nonflexible tools designed for other purposes [7] or software especially written and not accessible for the public. To overcome these issues, a more flexible software-tool is needed that allows defining the group of organisms individually for which specific COGs can be searched. Here we describe phylogenetic COG ranking (PCOGR), a platform independent software tool capable to rank all COGs with respect to a freely definable group of organisms versus a group of reference organisms.

## Implementation

PCOGR is written in PHP (v.4.3.3) including the domxml (v.20020815) plugin and runs on an openBSD (v.3.4) operating system at dmz.uni-wh.de in an apache (v.1.3.28) web-server environment. In addition, at the clients-side, HTML, javascript, and CSS are used.

Phylogenetic COG ranking (PCOGR) is an online-tool to analyze the microbial COG, or after clicking "Switch to PKOGR", to analyze the eukaryotic KOG database. PCOGR provides a means for determining the specificity of each COG with respect to the presence of sequences from organisms belonging to a predefined group (groupA) versus the absence of sequences from organisms belonging to a second predefined reference group (groupB). For that purpose, each of the organisms can be assigned to one of the two groups or defined to be ignored by the analysis. The software then calculates a specificity index S for every individual COG. The highest ranking COGs (large S) contain sequences from the most groupA-organisms whereas the most sequences from groupB-organisms are absent. To process S for each individual COG, the algorithm starts at S = 0, adds a constant A for each groupA-organism and subtracts a constant B for each groupB-organism being present in the COG under analysis with A = A_tot_/B_tot _and B = B_tot_/A_tot _where A_tot _is the total number of organisms belonging to groupA and B_tot _is the total number of organisms belonging to groupB. After all COGs have been processed in this way, all S-values are scaled to values between 0 and 1. Then, all COGs are output in the order of their specificity indexes S. In addition, a graphical representation shows the number of COGs as a function of their S-values in discrete intervals. The total number of intervals to be displayed can be specified by the user (default = 40 for PCOGR and 7 for PKOGR).

**Figure 1:**
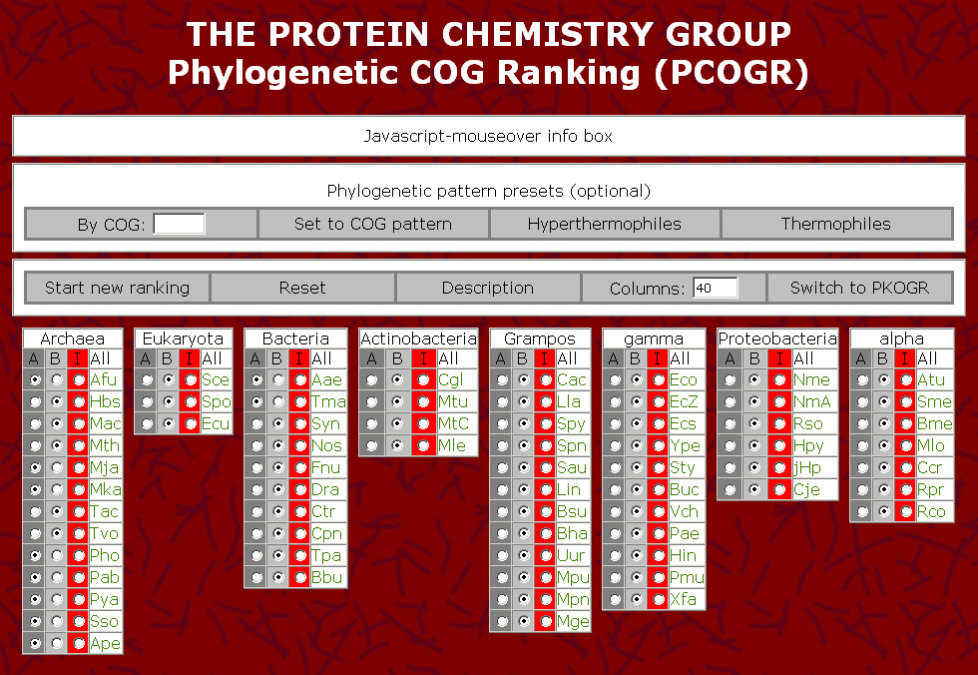
Screenshot of the parameter input section

**Figure 2:**
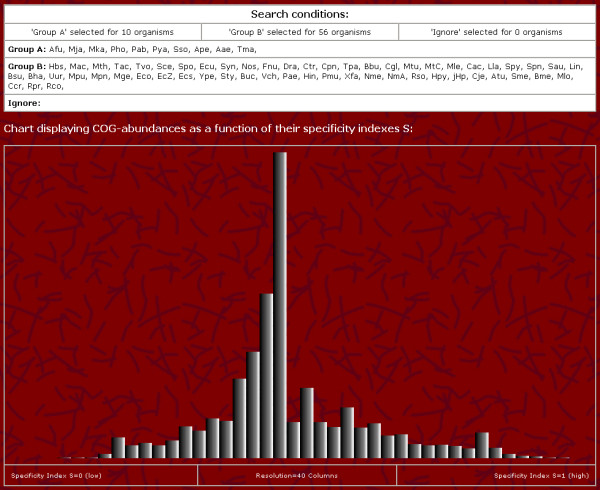
Screenshot of the graphical visualization

A Javascript-mouseover info box intuitively explains all functions of the graphical user interface of PCOGR. Furthermore, additional information about both, organisms and output COGs, are available by the implementation of links to  Figure 1, 2, and 3 show screenshots of the parameter input and output sections, respectively.

## Results and discussion

PCOGR allows detecting group-specific proteins by both ranking all COGs and graphically showing their distribution over their specificity indexes. The graphical representations can be interpreted as follows: If the two predefined groups are rather related, one expects a single peak in the middle of the graph, *i. e. *there are little or no proteins specific to one of the groups resulting in a specificity value of around 0.5 for most COGs. In contrast, if the two groups are rather distant, further maxima, either on the left, the right or on both sides become visible, *i. e. *there are group-specific proteins with S-values around 1 and/or S-values around 0. Even two single organisms can be compared by assigning the first to groupA, the second to groupB and ignoring all other organisms. For instance comparing the closely related *Escherichia coli *strains O157:H7 EDL933 and O157:H7 results in a prominent single peak in the middle of the graph whereas two further peaks on the edges become visible if two more distant organisms *e. g. Aquifex aeolicus *and *Saccharomyces cerevisiae *are compared.

Distance and relationship may be interpreted either in phylogenetic or in physiologic terms. To demonstrate that physiologic relevant differences in protein distributions indeed can be detected by PCOGR, two parameter-presets are selectable: (i) a specificity ranking of hyperthermophile-specific versus non-thermophile-specific proteins as published by Makarova et al. [5] and of thermophile-specific versus non-thermophile-specific proteins as described by Klinger et al. [6]. For the ranking according to Makarova et al., optimum growth temperatures of corresponding organisms belonging to groupA are all above 80°C and all other organisms are assigned to groupB. For the specificity ranking according to Klinger et al., the optimum growth temperature needed for an organism to be assigned to groupA is above 55°C instead of 80°C. The user will notice that for the two presets, there are two additional peaks, the first corresponding to COGs containing (hyper)thermophile-specific proteins, and the second peak corresponding to COGs containing mesophile-specific proteins.

A further attractive potential of PCOGR lies in the easy way to detect novel protein-protein interactions since physically interacting proteins should phylogenetically similarly be distributed [8]. Thus, if the phylogenetic pattern for a putative interacting protein target is known, a ranking with this pattern as the input will result in a ranking of potentially interacting candidates. To simplify such a procedure, the phylogenetic pattern of a certain COG defined by the user can automatically be assigned as the preset of a subsequent ranking. As an example, we performed a ranking choosing the phylogenetic pattern of COG2025 (electron transfer flavoprotein, alpha subunit). This ranking resulted in only two high-scoring outputs (specificity value S = 1): COG2025 (the target) and COG2086 (electron transfer flavoprotein, beta subunit) which is shown by x-ray crystallography to build a complex with the alpha subunit [9]. All following proteins have specificity values below 0.9 indicating the suitability of such a search for protein-protein interactions.

Not only protein-protein interactions can be detected but also enzymes involved in the same biochemical pathway as a certain target enzyme [8]. This possibility may be useful to find the biochemical function of yet uncharacterized proteins given that one or more catalysts of the same pathway are already characterized. For example, a search performed with the phylogenetic pattern of COG0135 (phosphoribosylanthranilate isomerase), an enzyme involved in the biosynthesis of L-tryptophan, results in four (COG0135, COG0159, COG0547, and COG0134) of the five enzymes involved in tryptophan biosynthesis at the top four places of the ranking. The beta subunit of tryptophan synthase is the only missing enzyme also involved in this pathway. A closer look reveals that this protein is assigned to two instead of one COGs (COG0133: rank 29 and COG1350: rank 1770). The latter COG is annotated as "predicted alternative tryptophan synthase beta-subunit (paralog of TrpB)". This double assignment may explain the absence of the beta subunit of tryptophan synthase from high-scoring proteins of the ranking.

Another attractive use of PCOGR can be to look for an alternative enzyme form catalyzing the same reaction but originated by non orthologous gene displacement (NOGD). Occurrence of NOGD in essential functions can be explored systematically by detecting complementary, rather than identical or similar, phylogenetic patterns [10]. A ranking performed with COG0588 (phosphoglycerate mutase 1) indeed resulted in COG3635 (predicted phosphoglycerate mutase, AP superfamily) at the seventh last rank (rank 4867 out of 4873) demonstrating that PCOGR is also well suited for such a purpose.

## Conclusions

With the online availability of PCOGR researchers can perform their own individual searches for group-specific proteins. This will not only allow a deeper insight into phylogenetic relationships of organisms or groups of organisms but also help to detect new highly group-specific proteins worth for isolation and further biochemical characterization. In addition, novel protein-protein interactions could be detected in silico, and this tool is also suitable to assign proteins of unknown function to partially known biochemical pathways. A further application lies in the search of alternate enzymes originated by convergent evolution.

## Availability and requirements

**Project name: **Phylogenetic COG ranking (PCOGR)

**Project home page: **

**Operating system(s): **Platform independent

**Programming language: **PHP, javascript, CSS and HTML

**Other requirements: **Web-browser capable to execute javascript

**License: **GNU General Public License

**Any restrictions to use by non-academics: **Contact authors

## Authors' contributions

FM carried out the software development and programming work. MK conceived of the study, and participated in its design and coordination and helped to draft the manuscript. All authors read and approved the final manuscript.

## Figures and Tables

**Figure 3:**
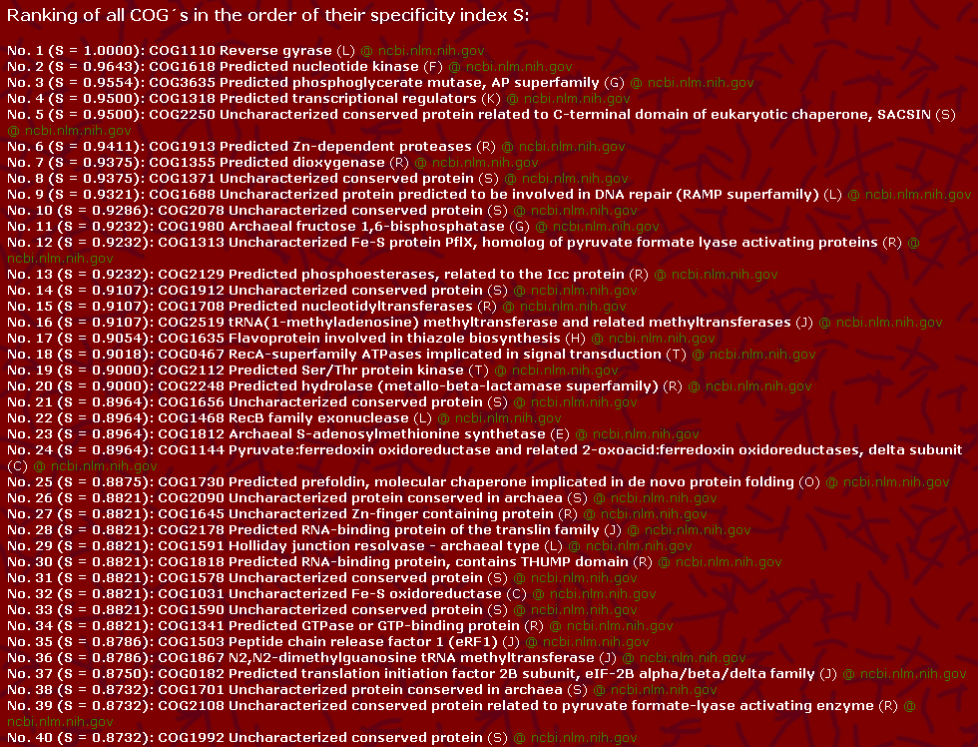
Partial screenshot of the COG-ranking
